# Soil Drench Treatment with ß-Aminobutyric Acid Increases Drought Tolerance of Potato

**DOI:** 10.1371/journal.pone.0114297

**Published:** 2014-12-09

**Authors:** Anita Sós-Hegedűs, Zsófia Juhász, Péter Poór, Mihály Kondrák, Ferenc Antal, Irma Tari, Brigitte Mauch-Mani, Zsófia Bánfalvi

**Affiliations:** 1 NARIC Agricultural Biotechnology Institute, P.O. Box 411, 2101, Gödöllő, Hungary; 2 Department of Plant Biology, University of Szeged, P.O. Box 654, 6701, Szeged, Hungary; 3 Faculty of Sciences, University of Neuchâtel, Neuchâtel, Switzerland; Chinese Academy of Sciences, China

## Abstract

The non-protein amino acid β-aminobutyric acid (BABA) is known to be a priming agent for a more efficient activation of cellular defence responses and a potent inducer of resistance against biotic and abiotic stresses in plants. Nevertheless, most of the studies on priming have been carried out in *Arabidopsis*. In potato, the effect of BABA was demonstrated only on biotic stress tolerance. We investigated the effect of BABA on the drought tolerance of potato and found that soil drenched with BABA at a final concentration of 0.3 mM improves the drought tolerance of potato. Water loss from the leaves of the primed plants is attenuated and the yield is increased compared to the unprimed drought-stressed plants. The metabolite composition of the tubers of the BABA-treated plants is less affected by drought than the tuber composition of the non-treated plants. Nitric oxide and ROS (reactive oxygen species) production is increased in the BABA-treated roots but not in the leaves. In the leaves of the BABA-treated plants, the expression of the drought-inducible gene *StDS2* is delayed, but the expression of *ETR1*, encoding an ethylene receptor, is maintained for a longer period under the drought conditions than in the leaves of the non-treated, drought-stressed control plants. This result suggests that the ethylene-inducible gene expression remains suppressed in primed plants leading to a longer leaf life and increased tuber yield compared to the non-treated, drought-stressed plants. The priming effect of BABA in potato, however, is transient and reverts to an unprimed state within a few weeks.

## Introduction

Crops often suffer from water shortage with a greater likelihood of drought stress in the future because of climate change and a decline in water resources for agriculture. Nevertheless, plants contain pre-existing and induced defences to mediate their adaptation to stress conditions. Over the past decades, increasing evidence has demonstrated that plants can be sensitised/primed for more efficient activation of cellular defence responses [1].

Most of the studies on priming have been carried out in *Arabidopsis*, in which several mutants have been identified with an altered priming phenotype. The *edr1* mutant was demonstrated to be constitutively primed suggesting that *EDR1*, encoding a putative mitogen-activated protein kinase kinase kinase (MAPKKK), functions as a negative regulator of priming [2]. The other genes identified in *Arabidopsis*, *NPR1*, *CPR1*, and *CPR5*, are associated with salicylic acid (SA)-mediated priming for enhanced defence-gene expression [3]. Mutations in genes encoding a cyclin-dependent kinase-like protein, a polyphosphoinositide phosphatase, or the abscisic acid (ABA) biosynthetic enzyme zeaxanthin epoxidase are compromised in induced resistance against *Hyaloperonospora parasitica* [4]. The primed state of *Arabidopsis* plants is transferred to their progeny and confers better protection from pathogen attack than in the descendants of unprimed plants [5].

The non-protein amino acid β-aminobutyric acid (BABA) is known to be a potent inducer of resistance in plants against microbial pathogens, nematodes, insects, and abiotic stresses. The mechanism of BABA-induced resistance (BABA-IR) involves an accumulation of pathogenesis-related (PR) proteins, ABA-dependent callose deposition, and jasmonic acid (JA)-dependent upregulation of phenylpropanoid-derived phenolic compounds [6]–[8]. Upon the application of salt and drought stress, the protection is correlated with an augmented expression of the SA-inducible *PR-1* and *PR-5* genes and the ABA-dependent drought-inducible *RAB-18* and *RD-29A* genes in *Arabidopsis* leaves [9].

Although IR mechanisms have been extensively studied in the model plant *Arabidopsis*, knowledge of IR in crops, including potato, is elusive. Nevertheless, Si-Ammour et al. [10] showed that spraying the susceptible potato cv. Bintje with BABA two days before inoculation with *Phytophthora infestans*, the potato late blight causative agent, resulted in a phenocopy of the incompatible interaction shown by the resistant potato cv. Matilda. Very recently, Floryszak-Wieczorek et al. [11] investigated how potato exposed to four different chemicals could activate nitric oxide (NO)-dependent events and facilitate more potent defence responses to a subsequent attack by *P. infestans*. The four chemicals sprayed on the leaves were BABA, γ-aminobutyric acid (GABA), laminarin, and 2,6-dichloroisonicotinic acid (INA). Arasimowicz-Jelonek et al. [12] detected the accumulation of 25 proteins specifically after the treatment with these four compounds, and 13 of these proteins accumulated in response to S-nitrosoglutathione reductase, a NO donor.

The positive effect of BABA on biotic stress tolerance of potato prompted us to investigate the effect of this chemical on abiotic stress tolerance of potato. In this report, the effects of BABA-drenched soil on drought tolerance of potato are presented.

## Methods

### Plant material, growth conditions, and BABA treatment

Potato (*Solanum tuberosum* L.) cv. Desirée was propagated vegetatively from cuttings on RM medium (MS medium without vitamins) [13] containing 2% (w/v) sucrose at 24°C under a light regime of 16 h at 170 *µ*mol photons m^−2^ s^−1^ light intensity and 8 h of darkness. Six-week-old plantlets from the *in vitro* culture were transferred to 1,000-ml pots containing A260 sterile soil (Stender AG, Schermbeck, Germany) and grown in a greenhouse at 20–28°C under long day conditions and 70% soil water content that was determined gravimetrically (g of water per g of soil) for a profile from 5 cm to 7 cm deep. Four weeks after being planted into the soil, the plants were treated by drenching their soil with different concentrations of BABA (Aldrich, Milwaukee, WI, USA, Cat. No. A44207, 20 ml/100 g soil with 70% water content) and the irrigation was halted. To test the effect of BABA on tuber yield and quality, seven dry cycles were created within a growing season. Water was withheld for 7–10 days in each cycle while the water content of the soil decreased to 30%. Between two dry cycles the plants were irrigated with an optimal amount of water for one week. Tubers were harvested from 4-month-old plants. When the plants were grown from tubers, the sprouting tubers were planted into 3,000-ml pots and treated in the same way as the plants grown from *in vitro* plantlets. An aerated hydroponic culture of potato was established in half-strength Hoagland's solution [14].

### Measurement of water content of leaves

The water content of the leaves was determined gravimetrically by measuring the fresh and dry weight of the leaves after drying for 16 h at 80°C. The water status of the plants was traced by determining the relative water content (RWC) according to the following equation:

RWC  =  (FW – DW) × 100/(SW – DW), where FW is the fresh weight, SW is the water saturated weight and DW is the dry weight of the leaves.

### RNA isolation and analysis

Total RNA was extracted from the leaves and tubers according to [15] and quantified using a NanoDrop spectrophotometer. Two micrograms of DNaseI-treated total RNA were reverse-transcribed with the High Capacity cDNA Reverse Transcription Kit (Applied Biosystems, Foster City, CA, USA). The cDNAs were diluted 10-fold and RT-PCR (reverse transcription-polymerase chain reaction) assays were performed with 49 primer pairs listed in S1 Table, including the *StDS2*-specific primers 5′-TGGTAATGAGGAAGGTGGCTA-3′and 5′-CAG CAC ACA AAG AGA GGT A-3′; the *ETR1-*specific primers 5′-GTT GCC TGC TGA CGA CTT GC-3′ and 5′- GCA CCG AAC TGC ACA AGA ACC-3′; and the *18S rRNA-*specific primers 5′-GGG CAT TCG TAT TTC ATA GTC AGA G-3′ and 5′-CGG TTC TTG ATT AAT GAA AAC ATC CT-3′.

### ROS and NO detection and quantification

For the detection of reactive oxygen species (ROS), the plant material was stained in the dark for 30 min at 37°C with 10 µM 2′,7′-dichlorodihydrofluorescein diacetate (H_2_DC-FDA) (Sigma-Aldrich, St. Louis, MO, USA) dissolved in 10 mM 2-(N-morpholino)ethanesulfonic acid (MES)/2-amino-2-hydroxymethyl-propane-1,3-diol TRIS/KCl buffer, pH 6.15 [16]. The fluorescence intensity was measured using filter set 10. The excitation occurred at 450–490 nm and the emission was detected with a 515–560 nm bandpass green filter using a Zeiss Axiovert 200 M-type fluorescence microscope (Carl Zeiss, Jena, Germany) equipped with a high-resolution digital camera (Axiocam HR; Carl Zeiss, Jena, Germany). The pixel intensity was determined in the 0.5 mm zones from the root apex and in the middle of the second leaves of the potato plants. The same camera settings were used for each digital image and the data were analysed using the Axiovision Rel. 4.8 software (Carl Zeiss, Jena, Germany).

NO (nitric oxide) was detected using 10 µM 4-amino-5-methylamino-2′,7′-difluorofluorescein (DAF-FM) (Sigma-Aldrich, St. Louis, MO, USA). For visualisation of NO, 1.5-cm-long root segments were incubated for 30 minutes in the dark in DAF-FM dissolved in 10 mM MES-TRIS/KCl buffer, pH 6.15 [17]. After staining, the samples were washed four times with the incubation buffer. The pixel intensity was determined in the same regions as the ROS detection. The microscope fields were chosen randomly.

For positive and negative controls, the generation and scavenging of ROS and NO were performed according to [18]. As a negative control, the root apices and leaf segments were incubated in a medium lacking H_2_DCF-DA or DAF-FM. After staining with specific dyes, the fluorescence caused by the addition of 10 µM H_2_O_2_ was effectively scavenged by 1 mM ascorbate, and by 100 U of catalase. The NO generated by the addition of 10 µM S-nitroso-N-acetylpenicillamine (SNAP) was diminished by 10 µM 2-(4-carboxyphenyl)-4,4,5,5-tetramethylimidazoline-1-oxyl-3-oxide (carboxy-PTIO) (data not shown).

### GC-MS analysis of polar metabolites from potato tuber extracts

Metabolites were analysed using gas chromatography-mass spectrometry (GC-MS). Tubers were harvested from the pots at full maturity. The tubers that were larger than 2 cm in diameter were selected, washed, and peeled. The piths were chopped with an electric blazer and stored at −70°C. Before extraction, the samples were ground to a fine powder in liquid nitrogen. The extraction was performed according to [19] using 125 mg of tuber powder. Ribitol was added to the samples as an internal standard. An aliquot of 150 *µ*L of the extract was dried with 30 *µ*L of ribitol (20 mg mL^−1^). For methoxyamination, 40 *µ*L of methoxyamine hydrochloride (MEOX) dissolved at 20 mg mL^−1^ in pyridine was added to the dried extract and agitated for 90 min at 37°C. N-methyl-N-(trimethylsilyl) trifluoroacetamide (MSTFA) was used for derivatisation (60 *µ*L, 30 min, 37°C). The samples were analysed in the split mode in a quadrupole-type GC-MS system (Finnigan Trace/DSQ, Thermo Electron Corp., Austin, TX, USA) equipped with a 30 m capillary column (Rxi-5 ms, 0.25 mm ID, 0.25 µm df, Restek, Bellefonte, PA, USA). Sample volumes of 1 µL were injected with a split ratio of 10∶1 using the hot needle technique. The injection temperature was 230°C, and the temperature of the interface and the ion source was set to 250°C. The carrier gas was helium, with a constant flow rate of 1 ml min^−1^. The temperature program included heating at 90°C for 2 min, followed by a 25°C min^−1^ oven temperature ramp to 165°C for 15 min. This ramp was followed by 6°C min^−1^ to 330°C. The system was temperature equilibrated for 2 min at 90°C prior to injection of the next sample. The detection was performed in total ion chromatogram (TIC) positive mode. Mass spectra were recorded at 0.8170 scans sec^−1^ with an *m/z* 50–650 scanning range.

The Thermo Scientific Xcalibur software was used for exporting the spectra and searching the NIST 11 mass spectral database. The NIST 11 is a fully evaluated collection of electron ionisation and mass spectra, with chemical and GC data, plus search software to identify unknown spectra. In addition, the sugars and amino acids were identified based on a comparison of the retention time and mass spectrum to an authentic standard that was analysed under identical conditions. Identification of the other class of compounds was based on the spectra searched against the retention index libraries downloadable from the Max-Planck Institute for Plant Physiology in Golm, Germany (http://gmd.mpimp-golm.mpg.de).

### Data analysis

Significant differences were established using *t*-test. Principal component analysis (PCA) was carried out using the Multibase Excel Add-Ins program, which can directly process Excel data (www.numericaldynamics.com).

## Results

### Elucidating the impact of BABA-priming on leaves under drought conditions

**Figure 1:**
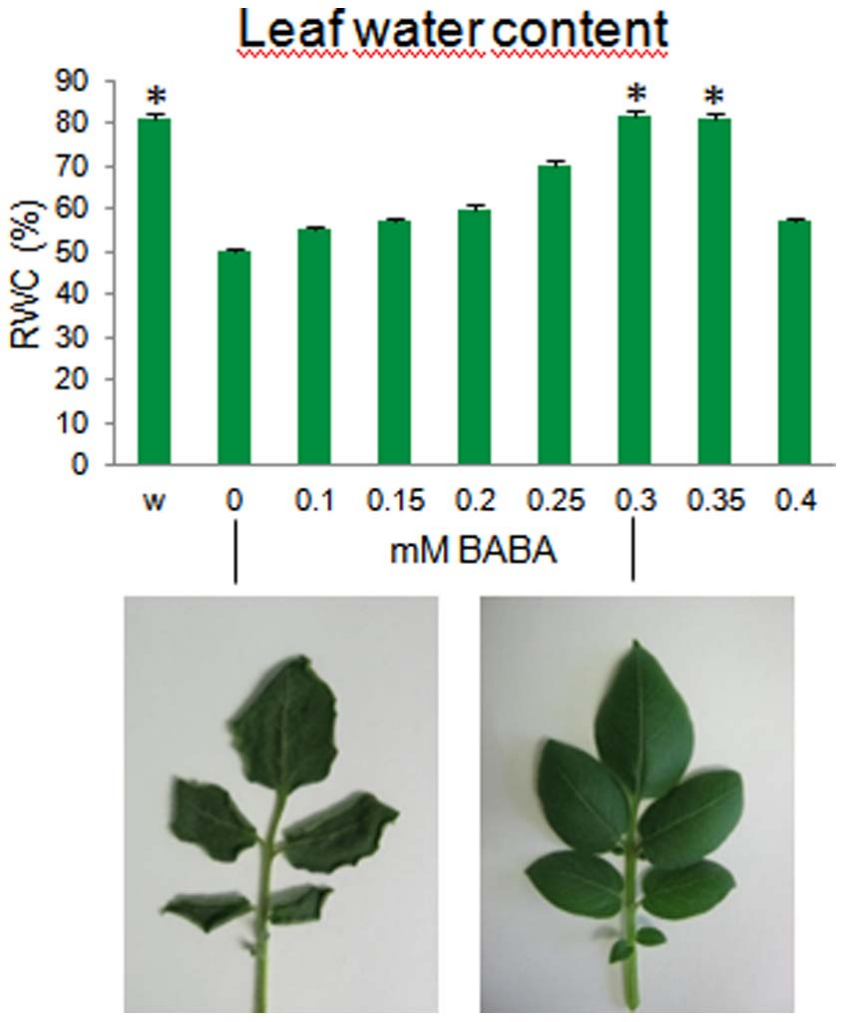
Efficiency of BABA treatments on water retention of the leaves 14 days after water withdrawal. The experiment began with four-week-old potted plants. The results are expressed as the mean ± SD of the data obtained from five compound leaves of five plants. Asterisks depict differences significant at *P*≤0.01 (*t*-test) compared to the non-treated drought-stressed control. w, well-watered; 0, non-treated, drought-stressed control.

Jakab et al. [9] reported that BABA can enhance abiotic stress tolerance of *Arabidopsis* at a concentration of 0.3 mM in the medium. The effect of BABA on abiotic stress tolerance of potato has not been previously tested. Therefore, in the first experiment, different concentrations of BABA were applied by drenching the soil (0, 0.1, 0.15. 0.2, 0.25, 0.3, 0.35, 0.4 mM final concentration in the soil) before the plants were subjected to dehydration in pots in a greenhouse. Fig. 1 shows that 14 days after water withdrawal, the leaves of the non-treated plants were wilted as their relative water content (RWC) was reduced from an average of 81.5% to 50.1%. In contrast, similar to that found in *Arabidopsis*, the plants treated with BABA at concentrations of 0.3 and 0.35 mM lost significantly less water from their leaves (RWC: 82.0 and 81.4%, respectively) than the non-treated control plants. BABA at a concentration of 0.4 mM, however, altered the phenotype of the plants as it restricted their growth and increased the green colour of the leaves.

### Transcriptional changes induced by BABA in leaves of drought-stressed plants

**Figure 2:**
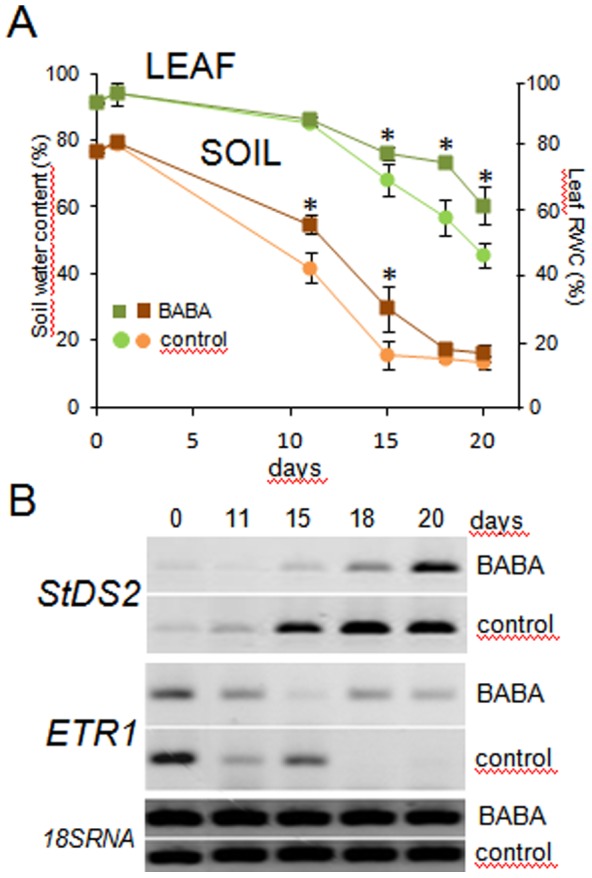
Changes in the RWC, soil water content (A) and gene expression (B) in the leaves of BABA-treated and non-treated control plants. Four-week-old potted plants were soil drenched with water or a solution of BABA at a final concentration of 0.3 mM in the soil. The irrigation was halted, and the soil water content and leaf RWC were assessed by testing three plants in parallel at each time point. The soil water content was determined gravimetrically for a profile from 5 cm to 7 cm deep. One compound leaf per plant was used for the RWC determination. RNA was isolated from three single leaves derived from three plants. The transcript levels were analysed using RT-PCR. The *18S RNA* gene with a constitutive level of expression was used as the internal control. The plant test was performed three times and similar results were obtained. The data of one representative experiment are shown in the figure. Asterisks depict differences significant at *P*≤0.01 (*t*-test) compared to the non-treated, drought-stressed control.

Drought stress induces transcriptional changes in leaves of plants [20]. Based on previous publications [9], [21] that assessed the effect of BABA treatment on gene expression in *Arabidopsis*, we selected 13 genes for an expression study of the leaves of the potato upon drought stress with and without 0.3 mM BABA treatment. Searches were performed for potato orthologs of *Arabidopsis* proteins and genes in various potato protein and nucleotide databases; however, sequences similar to only eight genes were detected. No potato ortholog to *PR-5*, *RAB-18*, or *RD-29A*, for example, could be identified. Hypothesising that BABA influences the expression of stress-inducible genes in potato, in addition to the selected eight *Arabidopsis* ortholog genes, the expression of 40 genes proved to be stress-inducible in different *Solanum* species and implicated in multiple pathways regulated by various hormones, i.e., ABA, SA, auxin, and ethylene, were tested (S1 Table). Reverse transcription-PCR (RT-PCR) analysis on total RNA preparations of the leaves at different time points after water withdrawal was carried out. The plant test was performed three times. The RT-PCR analysis was repeated with those genes that displayed altered expression in the leaves of the BABA-treated versus the non-treated drought-stressed plants in the first experiment. The soil moisture and relative water content (RWC) of the leaves were monitored in each experiment. A statistically significant difference in water uptake, as indicated by the water content of the soil of the BABA-treated and the non-treated plants, was observed on days 11 and 15 post water withdrawal (Fig. 2a). The RWC of the leaves was very similar until the 11^th^ day, when a sharp decrease in the RWC of the non-treated plants was detected, whereas the water loss of the BABA-treated plants was still moderate (Fig. 2a).

Two genes out of the 48 tested via RT-PCR showed consequent differences in gene expression between the BABA-treated and non-treated stressed plants. One of them was the drought-inducible gene *StDS2* [22]. In agreement with the improved water retention ability of the BABA-treated plants compared to the non-treated plants (Fig. 2a), the induction of *StDS2* expression was delayed in the leaves of the BABA-treated plants compared to the non-treated ones (Fig. 2b). The other gene with altered expression was *ethylene response 1* (*ETR1*), which encodes a membrane component that binds ethylene [23]. *ETR1* mRNA could be detected only in the leaves of the BABA-treated but not in the leaves of the non-treated plants 18 and 20 days after withholding the water supply (Fig. 2b).

### Effect of BABA on NO and ROS production in the roots

The statistically significant difference in water uptake of the BABA-treated and non-treated plants observed on days 11 and 15 after water withdrawal (Fig. 2a) suggested that the primary effect of BABA is on the root system of the plants. Floryszak-Wieczorek et al. [11] demonstrated that the spraying of potato leaves with priming agents, including BABA, increases NO production. This observation prompted us to investigate the NO concentration and the amount of ROS in the potato roots treated with 0.3 mM BABA in the hydroponic culture.

**Figure 3:**
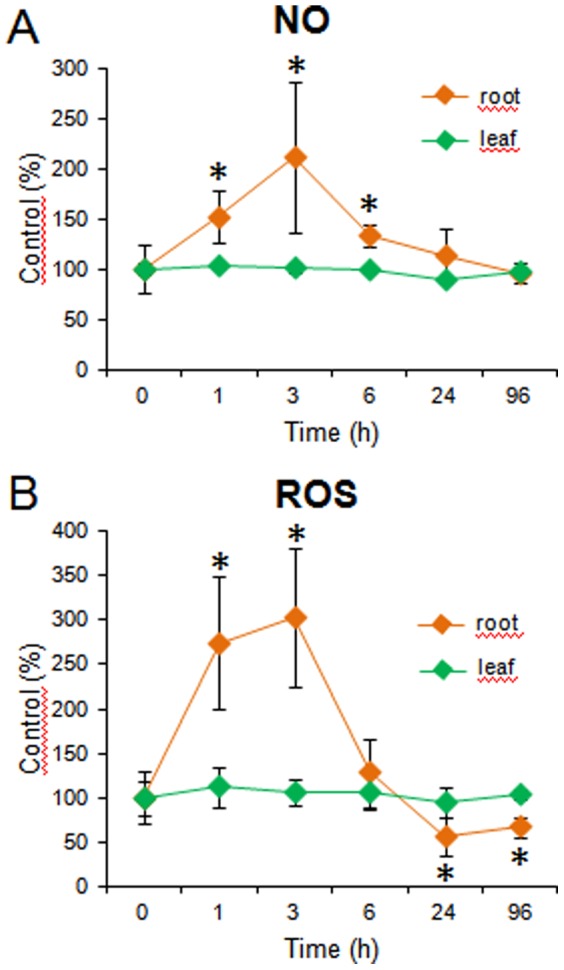
NO and ROS accumulation in the roots and leaves of potato plants grown in the presence of 0.3 mM BABA in hydroponic culture for 96 h. The accumulation of ROS and NO was visualised using the fluorescent dyes 2′,7′-dichlorodihydrofluorescein diacetate and 4-amino-5-methylamino-2′,7′-difluorofluorescein, respectively, and analysed in the root tips and second leaves. The results are expressed as the mean ± SD of the data obtained from nine plants. Asterisks depict differences significant at *P*≤0.01 (*t*-test) compared to the non-treated control (0 time).

Significantly increased ROS production was detected after one hour in the roots exposed to 0.3 mM BABA; however, after 24 hours, the ROS level was lower in the primed samples than in the controls. NO production peaked two hours later in the BABA-treated plants and after four days it declined to the control level (Fig. 3). These results suggest that BABA-induced NO and ROS production can contribute to the induction of the defence mechanisms in the roots of potato plants.

### Elucidating the impact of BABA-priming on tuber yield and quality under drought conditions

**Figure 4:**
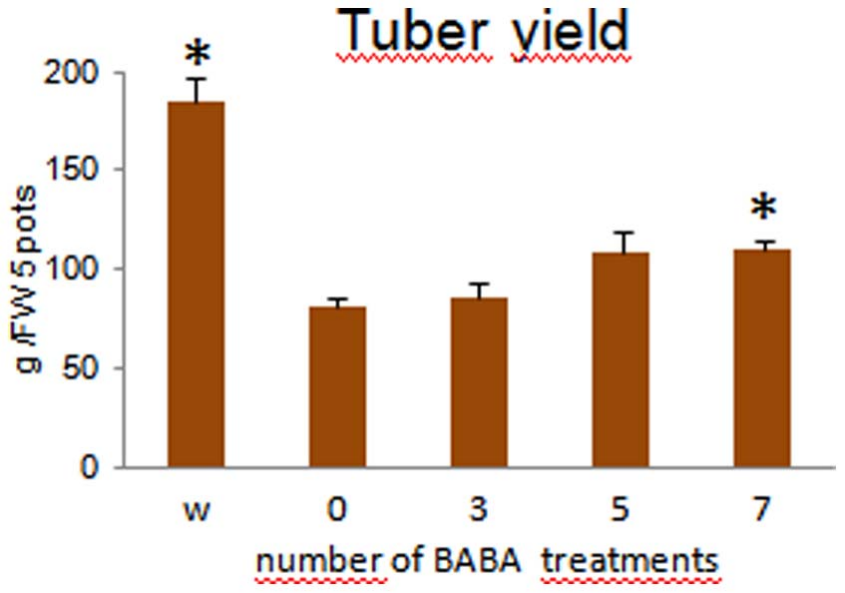
Effect of BABA on tuber yield of drought-stressed plants. The experiment began with four-week-old potted plants and was carried out in 15 parallels with seven drought and re-wetting cycles during the entire vegetation period. Soil drenching with BABA reached a final concentration of 0.3 mM. Tubers were collected in bulks from five pots. w, well-watered control; 0, water-treated, drought-stressed control; 3, treated with BABA before the 1^st^, 4^th^, and 7^th^ drought cycle; 5, treated with BABA before the 1^st^, 2^nd^, 3^rd^, 5^th^, and 7^th^ drought cycle; 7, treated with BABA before each drought cycle. Asterisks depict differences significant at *P*≤0.01 (*t*-test) compared to the non-treated, drought-stressed control.

Compared to other crops, the potato is considered drought sensitive and even short periods of drought stress can cause a significant reduction in tuber yield [24]. To test the effect of BABA on tuber yield and quality, seven drought periods with drying and re-wetting cycles were created within a 4-month growing season in the greenhouse (see Methods for details). The periodic drought stress reduced the yield of the non-treated control plants by approximately 60%. Drenching of the soil with 0.3 mM BABA before three or five drought cycles slightly elevated the yield. Nevertheless, a significant, 1.4-fold increase in yield compared to the non-treated control was achieved when BABA was applied before each drought cycle (Fig. 4).

**Figure 5:**
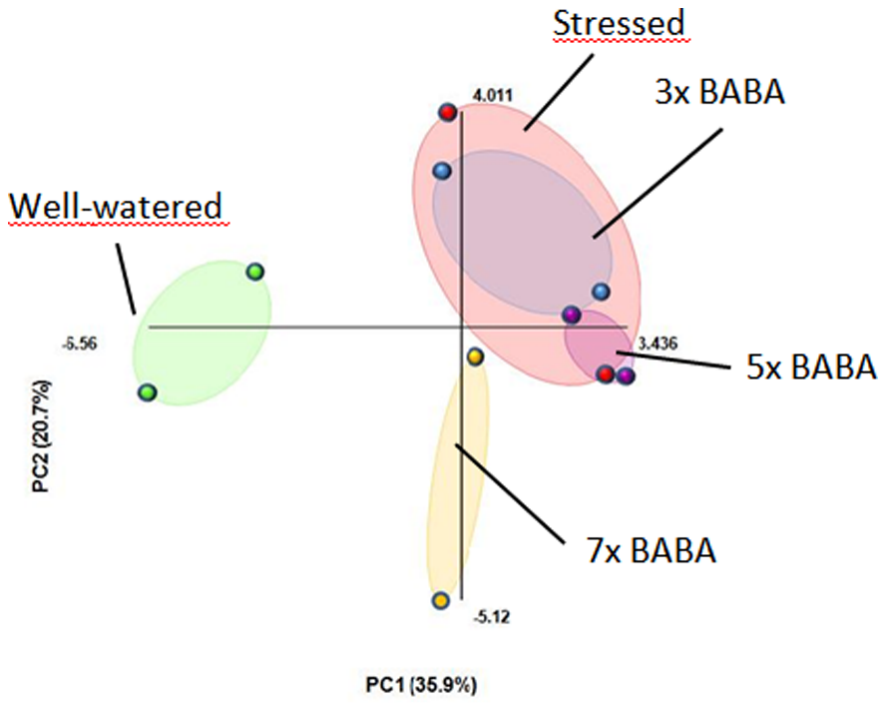
Principal component analysis of the metabolite composition of tubers of well-watered and drought-stressed plants without (0x BABA) and with BABA treatment (3x, 5x, 7x BABA – see **Fig. 4** legend for explanation). The tubers of five plants were analysed in two bulks, with each bulk containing 4-5 tubers. The data used for the PCA are presented in S2 Table.

The metabolite composition of tubers depends on the irrigation level of plants [25]. To obtain information on the effect of BABA treatment on the metabolite composition of the tubers of the drought-stressed plants, GC-MS analysis was performed. The pith of freshly harvested tubers derived from the experiment described above were analysed in two bulks, with each bulk containing 4–5 tubers. Thirty polar primary metabolites, including mostly carbohydrates, amino acids and sugar alcohols, were identified. Drought stress led to changes in the concentrations of most of the metabolites (S2 Table). The data were subjected to principal components analysis (PCA) to characterise the categories and define the major profiles of the metabolite changes. The PCA separated the tubers of the well-watered plants from the tubers of the non-treated drought-stressed plants (Fig. 5). This result was mainly due to the concentrations of proline, isocitric-, and malic acid, which were higher, and fructose, glucose, phenylalanine, and tryptophan concentrations, which were lower in the drought-stressed tubers than in the tubers of the well-watered plants. The PCA also showed that treatment of the plants with BABA before each drought cycle could alleviate the alterations in the metabolite composition elicited by a drought (Fig. 5).

Slaughter et al. [5] reported that the descendants of primed *Arabidopsis* plants exhibit resistance to biotic stress. To test the functionality of priming when the “next generation” is achieved by vegetative propagation of potato, pieces of the harvested tubers were stored at room temperature in the dark and monitored for sprouting every second week. No difference between the sprouting behaviour of the tubers was observed, as all of the tubers broke dormancy after twelve weeks. Ten tubers per category i.e., harvested from well-watered, non-treated, and 3-, 5-, or 7-time BABA-treated stressed plants, were planted in pots. The plants were grown under a periodic drought condition with seven drought and re-wetting cycles. No phenotypic differences between the plants were observed. The tuber yield of the plants also did not differ (data not shown), indicating that the primed state cannot be transferred by vegetative propagation.

## Discussion

Our results show that soil drenching with BABA at a final concentration of 0.3 mM improves drought tolerance of potato. Water loss of the leaves of the primed plants is attenuated and their yield is increased compared to the unprimed, drought-stressed plants (Figs. 1 & 4). Thus, the potato plants behave as *Arabidopsis* plants pre-treated with 0.3 mM BABA, which show a delayed onset of wilting and a reduced rate of water loss when subjected to dehydration [9].

Zimmerli et al. [21] detected statistically significant changes in the expression of 761 *Arabidopsis* genes using microarray analysis when the soil was drenched with a 0.25 mM BABA solution one day prior to harvesting the samples for RNA preparation. In that experiment, 46 genes were classified to the biological process category ‘response to stress’ including some genes associated with ABA or ethylene signalling and response. Based on these data, we expected to find several stress- and hormone-regulated potato genes with altered expression in the leaves of the BABA-treated plants. The transcript levels of 48 selected genes were tested using RT-PCR; however, no difference was detected in the expression of any of the genes in the leaves of the BABA-treated and water-treated plants one day after treatment. At this point, the plants were subjected to water deprivation. Although the RWC of the leaves was still quite high, the water content of the soil was substantially decreased by day 11 and the expression of *StDS2* was induced to a higher extent in the control plants than in the BABA-treated plants 15 days after water withdrawal (Fig. 2). The expression of *StDS2* is dehydration-specific in leaves, is not induced by cold, heat, salt, hypoxia or oxidative stresses and is independent of ABA [22]. The BABA priming delays the water-loss of leaves and thereby delays the expression of *StDS2*. In contrast, the expression of *ETR1* is down-regulated in unprimed but not in primed, drought-stressed plants. Ethylene has been considered to be the key hormone regulating leaf senescence, and drought may induce and accelerate leaf senescence [26]. The prolonged expression of *ETR1* elicited by BABA suggests that the regulation of the genes influenced by ETR1 is maintained in primed plants. The ethylene receptors are negative regulators [27]. In primed plants, the ethylene-inducible gene expression may remain suppressed and thus leads to a longer leaf life. In unprimed plants, the reduction in ethylene receptor levels can increase the sensitisation of plants to ethylene and accelerate leaf senescence.

BABA treatment decreased the water uptake of the plants. Therefore, we investigated how potato roots exposed to BABA activate the production of NO and ROS. We found that both the NO and ROS levels were elevated transiently in the root but not in the leaves of the BABA-treated plants (Fig. 3). Because the BABA treatment attenuated the water loss of the leaves, this result suggests that BABA triggers a root-shoot signal transduction mechanism that is mediated by NO and ROS. Research on the metabolism of these families of molecules suggests that there is a close relationship between redox homeostasis and the metabolism of reactive oxygen and nitrogen species that affects signal and transcriptional processes in cells under physiological and stress conditions [28]. Capone et al. [29] showed that the exposure of *Arabidopsis* roots to H_2_O_2_ or NO resulted in a rapid activation of protein kinases in the shoots that exhibited mitogen-activated protein kinase (MAPK) properties. MAP kinases, along with phosphatases, function as on/off signal switchers to regulate the activity of many downstream targets, such as cytoskeletal proteins in the cytosol or transcription factors in the nucleus, to control cell signalling in plant adaptation to environmental oscillations [30]. We hypothesise that a mechanism similar to that found in *Arabidopsis* exists in potato and that MAPKs are also involved in root-shoot communication in this species.

The priming effect of BABA on potato is transient, and the plant reverts to an unprimed state within a few weeks, as indicated by the significant increase in tuber yield (Fig. 4) and quality (Fig. 5) compared to the non-treated, drought-stressed control that could be achieved only by drenching the soil with BABA before each drought cycle. Furthermore, the priming effect of BABA could not be retained in the plants grown from tubers. Numerous reports exist regarding the transgenerational priming via seeds, especially those mediated by the up-regulation of the SA- or JA-related signalling pathways [31]. These reports suggest that the molecular mechanisms underlying induced defence inheritance depend on the stress or the priming agent to which the parental lines were exposed. Because Slaughter et al. [5] detected inheritance by the seeds of BABA-treated plants, the priming agent could not be the reason underlying the detected difference in the duration of the primed state between *Arabidopsis* and potato. The relatively quick reversion of the primed state in our experiment might be due to the type of stress, i.e., abiotic in potato versus biotic in *Arabidopsis*, or the type of reproduction, i.e., asexual in potato versus sexual in *Arabidopsis*.

## Conclusions

In summary, we can conclude that soil drenching by BABA improves drought tolerance of potato. This conclusion is based on the following findings: (1) the plants treated with BABA at a concentration range of 0.3–0.35 mM lost significantly less water from their leaves than the non-treated control plants; (2) a 1.4-fold increase in yield compared to the non-treated control was achieved when BABA was applied at a concentration of 0.3 mM before each drought cycle; (3) the metabolite composition of the tubers of the BABA-treated plants was less affected by drought than the tuber composition of the non-treated plants. Out of 48 tested genes implicated in stress responses and hormone-regulated pathways, the soil drenching by BABA altered the expression of only two genes in the leaves under the drought conditions: the drought-inducible gene, *StDS2*, and the ethylene receptor gene, *ETR1*. Treatment of the roots with BABA transiently activated NO and ROS production in the roots but not in the leaves, suggesting that the signal generated by BABA in the roots is transduced before being transported to the foliage. The priming effect of BABA in potato is transient and the plant reverts to an unprimed state within a few weeks. Recently, Sani et al. [32] demonstrated that the hyperosmotic priming of *Arabidopsis* seedlings, which also diminishes over time, establishes a somatic memory accompanied by specific changes of the epigenome and preferentially targets transcription factors. The identities of the transcription factors targeted by BABA in potato are of great interest.

## Supporting Information

S1 Table **Primers used in RT-PCR analysis and reference list of genes tested for expression in the leaves of potato plants.**
(DOC)

S2 Table **Metabolite data file including a checklist, reporting list and data matrix.** Reporting of the metabolite data follows the guideline of [33].
(XLS)

## References

[1] Prime-A-Plant Group: Conrath U, Beckers GJ, Flors V, García-Agustín P, Jakab G, et al (2006) Priming: getting ready for battle. Mol Plant-Microbe Interact 19:1062–1071. 17022170 10.1094/MPMI-19-1062

[2] Frye CA, Tang D, Innes RW (2001) Negative regulation of defense responses in plants by a conserved MAPKK kinase. Proc Natl Acad Sci U S A 98:373–378. 11114160 10.1073/pnas.98.1.373 PMC14597

[3] Conrath U, Pieterse CMJ, Mauch-Mani B (2002) Priming in plant-pathogen interactions. Trends Plant Sci 7:210–216. 11992826 10.1016/s1360-1385(02)02244-6

[4] Ton J, Jakab G, Toquin V, Flors V, Iavicoli A, et al (2005) Dissecting the ß-aminobutyric acid-induced priming phenomenon in *Arabidopsis* . Plant Cell 17:987–999. 15722464 10.1105/tpc.104.029728 PMC1069713

[5] Slaughter A, Daniel X, Flors V, Luna E, Hohn B, et al (2012) Descendants of primed Arabidopsis plants exhibit resistance to biotic stress. Plant Physiol 158:835–843. 22209872 10.1104/pp.111.191593 PMC3271771

[6] Jakab G, Cottier V, Toquin V, Rigoli G, Zimmerli L, et al (2001) β-aminobutyric acid-induced resistance in plants. Eur J Plant Pathol 107:29–37.

[7] Ton J, Mauch-Mani B (2004) β-amino-butyric acid-induced resistance against necrotrophic pathogens is based on ABA-dependent priming for callose. Plant J 38:119–130. 15053765 10.1111/j.1365-313X.2004.02028.x

[8] Hamiduzzaman MM, Jakab G, Barnavon L, Neuhaus J-M, Mauch-Mani B (2005) β-aminobutyric acid-induced resistance against downy mildew in grapevine acts through the potentiation of callose formation and jasmonic acid signaling. Mol Plant-Microbe Interact 18:819–829. 16134894 10.1094/MPMI-18-0819

[9] Jakab G, Ton J, Flors V, Zimmerli L, Métraux JP, et al (2005) Enhancing *Arabidopsis* salt and drought stress tolerance by chemical priming for its abscisic acid responses. Plant Physiol 139:267–274. 16113213 10.1104/pp.105.065698 PMC1203376

[10] Si-Ammour A, Mauch-Mani B, Mauch F (2003) Quantification of induced resistance against *Phytophthora* species expressing GFP as a vital marker: beta-aminobutyric acid but not BTH protects potato and Arabidopsis from infection. Mol Plant Pathol 4:237–248. 20569384 10.1046/j.1364-3703.2003.00168.x

[11] Floryszak-Wieczorek J, Arasimowicz-Jelonek M, Milczarek G, Janus L, Pawlak-Sprada S, et al (2012) Nitric oxide-mediated stress imprint in potato as an effect of exposure to a priming agent. Mol Plant-Microbe Interact 25:1469–1477. 22835274 10.1094/MPMI-02-12-0044-R

[12] Arasimowicz-Jelonek M, Kosmala A, Janus L, Abramowski D, Floryszak-Wieczorek J (2013) The proteome response of potato leaves to priming agents and S-nitrosoglutathione. Plant Sci 198:83–90. 23199689 10.1016/j.plantsci.2012.10.004

[13] Murashige T, Skoog F (1962) A revised medium for rapid growth and bioassays with tobacco tissue culture. Physiol Plantarum 15:473–497.

[14] Hoagland DR, Arnon DI (1950) The water-culture method for growing plants without soil. Cal Agricult Exp Stat Circ 347:1–32.

[15] Stiekema WJ, Heidekamp F, Dirkse WG, van Beckum J, de Haan P, et al (1988) Molecular cloning and analysis of four potato tuber mRNAs. Plant Mol Biol 11:255–269. 24272339 10.1007/BF00027383

[16] Allan AC, Fluhr R (1997) Two distinct sources of elicited reactive oxygen species in tobacco epidermal cells. Plant Cell 9:1559–1572. 12237396 10.1105/tpc.9.9.1559 PMC157033

[17] Kolbert Z, Pető A, Lehotai N, Feigl G, Ördög A, et al (2012) *In vivo* and *in vitro* studies on fluorophore specificity. Acta Biol Szeged 56:37–41.

[18] Neill S, Desikan R, Clarke A, Hurst RD, Hancock JT (2002) Hydrogen peroxide and nitric oxide as signalling molecules in plants. J Exp Bot 53:1237–1247. 11997372

[19] Schauer N, Zamir D, Fernie AR (2005) Metabolic profiling of leaves and fruit of wild species tomato: a survey of the *Solanum lycopersicum* complex. J Exp Bot 56:297–307. 15596477 10.1093/jxb/eri057

[20] Tran LS, Nakashima K, Shinozaki K, Yamaguchi-Shinozaki K (2007) Plant gene networks in osmotic stress response: from genes to regulatory networks. Methods Enzymol 428:109–128. 17875414 10.1016/S0076-6879(07)28006-1

[21] Zimmerli L, Hou BH, Tsai CH, Jakab G, Mauch-Mani B, et al (2008) The xenobiotic ß-aminobutyric acid enhances Arabidopsis thermotolerance. Plant J 53:144–156. 18047473 10.1111/j.1365-313X.2007.03343.x

[22] Dóczi R, Csanaki C, Bánfalvi Z (2002) Expression and promoter activity of the desiccation-specific *Solanum tuberosum* gene, *StDS2* . Plant Cell Environ 25:1197–1203.

[23] Chang C, Kwok SF, Bleecker AB, Meyerowitz EM (1993) Arabidopsis ethylene-response gene *ETR1*: similarity of product to two-component regulators. Science 262:539–544. 8211181 10.1126/science.8211181

[24] Loon van (1981) The effect of water stress on potato growth, development and yield. Am Potato J 58:51–69.

[25] Maggio A, Carillo P, Bulmetti GS, Fuggi A, Barbieri G, et al (2008) Potato yield and metabolic profiling under conventional and organic farming. Eur J Agronomy 28:343–350.

[26] Zhang H, Zhou C (2013) Signal transduction in leaf senescence. Plant Mol Biol 82:539–545. 23096425 10.1007/s11103-012-9980-4

[27] Wang F, Cui X, Sun Y, Dong CH (2013) Ethylene signaling and regulation in plant growth and stress responses. Plant Cell Rep 32:1099–1109. 23525746 10.1007/s00299-013-1421-6

[28] Baudouin E (2011) The language of nitric oxide signalling. Plant Biol (Stuttg) 13:233–242. 21309969 10.1111/j.1438-8677.2010.00403.x

[29] Capone R, Tiwari BS, Levine A (2004) Rapid transmission of oxidative and nitrosative stress signals from roots to shoots in Arabidopsis. Plant Physiol Biochem 42:425–428. 15191746 10.1016/j.plaphy.2004.03.005

[30] Moustafa K, AbuQamar S, Jarrar M, Al-Rajab AJ, Trémouillaux-Guiller J (2014) MAPK cascades and major abiotic stresses. Plant Cell Rep 33:1217–1225. 24832772 10.1007/s00299-014-1629-0

[31] Holeski LM, Jander G, Agrawal AA (2012) Transgenerational defense induction and epigenetic inheritance in plants. Trends Ecol Evol 27:618–626. 22940222 10.1016/j.tree.2012.07.011

[32] Sani E, Herzyk P, Perrella G, Colot V, Amtmann A (2013) Hyperosmotic priming of Arabidopsis seedlings establishes a long-term somatic memory accompanied by specific changes of the epigenome. Genome Biol 14:R59. 23767915 10.1186/gb-2013-14-6-r59 PMC3707022

[33] Fernie AR, Aharoni A, Willmitzer L, Stitt M, Tohge T, et al (2011) Recommendations for reporting metabolite data. Plant Cell 23:2477–2482. 21771932 10.1105/tpc.111.086272 PMC3226225

