# Supplementary material for: Soil Drench Treatment with ß-Aminobutyric Acid Increases Drought Tolerance of Potato
Source: PLoS One. 2014 Dec 9;9(12):e114297. doi: 10.1371/journal.pone.0114297 (PMC4260862; doi:10.1371/journal.pone.0114297)
Supplement: S1 Table — Primers used in RT-PCR analysis and reference list of genes tested for expression in the leaves of potato plants. (DOC) [file pone.0114297.s001.doc]

**Table S1**

Genes tested for expression by RT-PCR

| **Acc. No.** | **Gene** | **Species** | **Primers** | **Reference^a^** |
| --- | --- | --- | --- | --- |
| AY615281 | *NPR1*-like | *S. tuberosum* | 5'- GAGCTTCTCACTCATTGCGTT -3'  5'- CGCATCTCTCT TC AAAACA T -3' | Pajerowska, Parker & Gebhardt (2005) |
| AJ242551 | *OPR* | *S. lycopersicum* | 5’- GCTTTGGGACTTTACATGGTG -3’  5’- TCATCGGTACAAGGCTTTCAG -3’ | Strassner et al. (1999) |
| AK325973 | *Rpd1*-like | *S.lycopersicum* | 5’- TGAAAGAGACCCGCGATTAAC -3’  5’- CGCCACCCTTTTCTCTATACTC -3’ | Konishi & Sugiyama (2006) |
| PGSC0003DMT400045156 | *PYR1* | *S. phureja* | 5'- GAATCAACTCGGGCCAATG -3'  5'- GAAGTCTTCACCTACCGAAC G -3' | Peterson et al. (2010) |
| PGSC0003DMT400041080 | *PYL4* | *S. phureja* | 5'- ACCCTCAA CGTACAAACAC -3'  5'- GAAGAGTGCCTACGTTACCG -3' | Lackman et al. (2011) |
| X63103 | PAL-1 | *S. tuberosum* | 5'- TGCACAAGTTGCATCCAT T -3'  5'- AAGAGCACCACCATTTTTGG -3' | Henriquez et al. (2012) |
| GI:82400145 | *H2B* | *S. tuberosum* | 5′- GGGAAGAAGCTACCAAAGGA -3’  5′- GGGCATTCGTATTTCATAGTCAGAG -3′ | Floryszak-Wieczorek et al. (2012) |
| AJ320154 | *StDS2* | *S. tuberosum* | 5’- TGGTAATGAGGAAGGTGGCTA -3’  5’- CAGCACACAACAAAGAGAGGTA -3’ | Dóczi, Csanaki & Bánfalvi (2002) |
| HM988989 | *StABF1* | *S. tuberosum* | 5’- CAACAGAACCATCAACCACAG -3’  5’- CAACCATCCCTACCCTCATTC -3’ | Muñiz García et al. (2012) |
| EF093186 | *StZFP1* | *S. tuberosum* | 5’- TTCCACAAGCGGTAATATCTCTG -3’  5’- AGTTTACCTTCGTAGTGGCG -3’ | Tian et al. (2010) |
| EF596909 | *GST* | *S. tuberosum* | 5’- TTACCCTAGCTGATTTGCACC -3’  5’- ACCCAAGCACTAACATGAGG -3’ | Vasquez-Robinet et al. (2008) |
| AF261142 | *TRX H* | *S. tuberosum* | 5’- GCACTCCGTACTTCACCTTC -3’  5’- AGGAGCGTGTTTGAAGAGTG -3’ | Vasquez-Robinet et al. (2008) |
| U76610 | *ci21A* | *S. tuberosum* | 5’- TGTATGGAGTGTTGTCGTGTT -3’  5’- CCATACAACCTCTTTACATCACAC -3’ | Schneider, Salamini & Gebhardt (1997) |
| HM013965 | *ZEP* | *S. tuberosum* | 5’- TGTAGGAGCTTGGAAAATGGG -3’  5’- AAATCACCGTTAACCCCAGAG -3’ | Wolters et al. (2010) |
| AJ250136 | *StPR-1* | *S. tuberosum* | 5’- GCATCCCGAGCACAAAATTATG -3’  5’- TTGTAGTTTGGCTTCTCCCC -3’ | van't Klooster et al. (1999) |
| AB062138 | *StMPK* | *S. tuberosum* | 5’- GCACATCCTTACCTAGCATCG -3’  5’- GCTCCTTCATCTGTTCCTCTG -3’ | Katou et al. (2005) |
| NM_001246967 | *TPS1* | *S. lycopersicum* | 5’- ATTCTGGATGCTCGTTCA -3’  5’- GATGAAATCGCAGACTTACA -3’ | Gómez et al. (2010) |
| NM_001247431 | *SlMPK3* | *S. lycopersicum* | 5’-GATCGGATCCATGGTTGATGCTAATATGGG-3’  5’-GATCCTCGAGTTAAGCATATTCAGGATT  CAACG-3’ | Stulemeijer, Stratmann & Joosten (2007) |
| NM_001247373 | *GGPPS* | *S. lycopersicum* | 5’- GACCCACCTAAGATCCATGAAG -3’  5’- GCATTTCCTTGGTTTCCCC -3’ | Loyola et al. (2012) |
| GQ222384 | *NCED* | *S. lycopersicum* | 5’- GTACTTCCTATTCTCGGCAGAC -3’  5’- ACGTATTTCTCAGTGATCGCG -3’ | Loyola et al. (2012) |
| FJ654265 | *NAC6* | *S. lycopersicum* | 5’- AACGAAAGTTGATCGATACAAGTAA -3’  5’- AAGGATACTATATGAACAAACACCC -3’ | Huang et al. (2012) |
| FJ654265 | *WRKY1* | *S. lycopersicum* | 5'- CGGAACTCAAAGAGACGGAAG -3'  5’- CATTGACTACATCCACTTCACTGC -3’ | Huang et al. (2012) |
| FJ654264 | *WRKY2* | *S. lycopersicum* | 5’- CACCTACGAAGGGAAGCACA -3’  5’- GAAGGTTGCCTGTCAAATCG -3’ | Huang et al. (2012) |
| Solyc06g066370.2.1 | *WRKY31* | *S. lycopersicum* | 5’- ACAACCTATGAAGGGAAGCACA -3’  5’- AGGGTGCTCCCATTTCAGAC -3’ | Huang et al. (2012) |
| AF043084 | *ETR1* | *S. lycopersicum* | 5’- GTTGCCTGCTGACGACTTGC -3’  5’- GCACCGAACTGCACAAGAACC -3’ | Santisree et al. (2011) |
| AY600437 | *ETR3* | *S. lycopersicum* | 5’- CGCAGATCAGGTTGCTGTCG -3’  5’- TGGGCGTTCTCATTTCATGG -3’ | Santisree et al. (2011) |
| AF118843 | *ETR4* | *S. lycopersicum* | 5’- AGCCAGAGGGGACCATGTTG -3’  5’- CCCAAGAACGACAGCCATGC -3’ | Santisree et al. (2011) |
| AY600439 | *ETR5* | *S. lycopersicum* | 5’- ATTCGGATGCCACTGCTTCG -3’  5’- ATTCGGATGCCACTGCTTCG -3’ | Santisree et al. (2011) |
| AY600440 | *ETR6* | *S. lycopersicum* | 5’- CCCCTGCTCCTCCAACATACG -3’  5’- ATCCGACAATCACAGCCATGC -3’ | Santisree et al. (2011) |
| AJ937282 | IAA9 | *S. lycopersicum* | 5’- GATTGTTCGTCGGTGGACAGC -3’  5’- AAGGGTGCAAGGGGAAGAGC -3’ | Santisree et al. (2011) |
| AF022014 | IAA3 | *S. lycopersicum* | 5’- GCATGGATGGAGCACCTTATTTG -3’  5’- TGGTACATCACCAACAAGCATCC -3’ | Santisree et al. (2011) |
| SGN-E1245095 | MCA1 | *S. lycopersicum* | 5’- GCCCTCCTGCTGATCTGTGC -3’  5’- CCCAATTAGCCTCACCGCATC -3’ | Santisree et al. (2011) |
| CK269661 | StPIN1a | *S. tuberosum* | 5’- CCAGTAAGGAATCTCACACCC -3’  5’- TCTTTCTGATGCTGGTCTTGG -3’ | Vieten et al. (2005) |
| AK324839 | SCR-like | *S. lycopersicum* | 5’- AACCTAACTTACACAGACGCC -3’  5’- TTTGGCGGTTTCTATCTGGAG -3’ | Cui, Hao & Kong (2012) |
| AC226596 | SHR-like | *S. lycopersicum* | 5’- TCCACGACAAGAATATGCCG -3’  5’- TCCACGACAAGAATATGCCG -3’ | Wang et al. (2011) |
| EU664445 | *AGL11* | *S. tuberosum* | 5’- TCGCAGCTTAGTCAATAGTCATC -3’  5’- ACTCGCCCTTGTTTCAGTAAC -3’ | Favaro et al. (2003) |
| XM_004238748 | *ERD3* | *S. lycopersicum* | 5’- TGTTACCTCATTTCCGTGGC -3’  5’- ACGTTACCCTCATATTGAACCC -3’ | Taji et al. (1999) |
| AY205084 | *SUS3* | *S. tuberosum* | 5’- CTCTCCAAAAAGTCCTGAGGAAGGCT -3’  5’- TGCCGAGTATGACGTTTCTGGGG -3’ | Fu & Park (1995) |
| AF133814 | *ferritin* | *S. tuberosum* | 5’- CGTGGCTGATCGAAACAATG -3’  5’- ATGTCCTTTTCCAACCCTCC -3’ | Ravet et al. (2009) |
| HM988989 | *ABF3* | *S. tuberosum* | 5’- CAACAGAACCATCAACCACAG -3’  5’- CAACCATCCCTACCCTCATTC -3’ | Yoshida et al. (2010) |
| XM_004230884 | *RD2* | *S. lycopersicum* | 5’- ATTGTGGAAGGAGATGCTGG -3’  5’- ATACTCAGCGACACTTCCTTG -3’ | Klopffleisch et al. (2011) |
| S70186 | *HSP23* | *S. tuberosum* | 5’- ACTCTGCCGCTTTCTATTGC -3’  5’- TCTTCCGCTCTCCACTCA -3’ | Lee et al. (2012) |
| X69762 | *rbcS2c* | *S. tuberosum* | 5’- GGCCACCAATTAACATGAAGAAGT -3’  5’- AAGGAAAACGGAAAATGCCAACAG -3’ | Fritz et al.(1993) |
| XM_006355515 | *SRG1*-like | *S. tuberosum* | 5’- TCCACCATGTCCACAACCAGACA -3’  5’- CCTCATTGGTGATTCTCATGGTGTCCA -3’ | Ouyang et al. (2007) |
| NM_001288060 | *Remorin* | *S. tuberosum* | 5’- TCCTGCAGAAGAAAAAGAAAAACC -3’  5’- CAAAAATGTACTCCCCAATCACTA -3’ | Reymond et al. (1996) |
| XM_006340656 | *HistonH1-*like | *S. tuberosum* | 5’- CTGAGGCTGGAAAGAAGGAGACTACA -3’  5’- AACGCCTTAATTTGCAGAGACACAGA -3’ | Jayawardene & Riggs (1994) |
| NM_001287934 | *CAT1* | *S. tuberosum* | 5’- ATACGACACCCCGTTTCTTG -3’  5’- AGGGCACGACTTAGCATCAC-3’ | Wu c Shah (1995) |
| NM_001288213 | *POTM1-1* | *S. tuberosum* | 5’- GAGTCCAGTTGAAGCGAATAGAG-3’  5’- GTGGCAAAACAAATGTAGATGAGT-3’ | Kang & Hannapel (1995) |
| GI:511154 | *18S rRNA* | *S. tuberosum* | 5′- GGGCATTCGTATTTCATAGTCAGAG-3′  5′- CGGTTCTTGATTAATGAAAACATCCT-3′ | Floryszak-Wieczorek et al. (2012) |

**^a^**References are for the function of the genes.

References

Cui H, Hao Y, Kong D (2012) SCARECROW has a SHORT-ROOT-independent role in modulating the sugar response. Plant Physiol 158: 1769-1778.

Dóczi R, Csanaki C, Bánfalvi Z (2002) Expression and promoter activity of the desiccation-specific *Solanum tuberosum* gene, *StDS2*. Plant Cell Environ 25: 1197–1203.

Favaro R, Pinyopich A, Battaglia R, Kooiker M, Borghi L, Ditta G, Yanofsky MF, Kater MM, Colombo L (2003) MADS-box protein complexes control carpel and ovule development in Arabidopsis. Plant Cell 15: 2603-2611.

Floryszak-Wieczorek J, Arasimowicz-Jelonek M, Milczarek G, Janus L, Pawlak-Sprada S, Abramowski D, Deckert J, Billert H (2012) Nitric oxide–mediated stress imprint in potato as an effect of exposure to a priming agent. Mol Plant-Microb Interact 25: 1469-1477.

Fritz CC, Wolter FP, Schenkemeyer V, Herget T, Schreier PH (1993) The gene family encoding the ribulose-(1,5)-bisphosphate

carboxylase/oxygenase (Rubisco) small subunit of potato. Gene 137: 271-274.

Fu H, Park WD (1995) Sink- and vascular-associated sucrose synthase functions are encoded by different gene classes in potato. Plant Cell 7: 1369-1385.

Gómez LD, Gilday A, Feil R, Lunn JE, Graham IA (2010) AtTPS1-mediated trehalose 6-phosphate synthesis is essential for embryogenic and vegetative growth and responsiveness to ABA in germinating seeds and stomatal guard cells. Plant J 64: 1-13.

Henriquez MA, Wolski EA, Molina OI, Adam LR, Andreu AB, Daayf F (2012) Effects of glucans and eicosapentaenoic acid on differential regulation of phenylpropanoid and mevalonic pathways during potato response to *Phytophthora infestans*. Plant Physiol Biochem 60: 119-128.

Huang S, Gao Y, Liu J, Peng X, Niu X, Fei Z, Cao S, Liu Y (2012) Genome-wide analysis of WRKY transcription factors in *Solanum lycopersicum*. Mol Genet Genom 287: 495-513.

Jayawardene N, Riggs CD (1994) Molecular cloning, sequence analysis and differential expression of an intron-containing gene encoding tomato histone H1. Eur J Biochem 223: 693-699.

Kang SG, Hannapel DJ (1995) Nucleotide sequences of novel potato (*Solanum tuberosum* L.) MADS-box cDNAs and their expression in vegetative organs. Gene 166: 329-330.

Katou S, Yoshioka H, Kawakita K, Rowland O, Jones JD, Mori H, Doke N (2005) Involvement of PPS3 phosphorylated by elicitor-responsive mitogen-activated protein kinases in the regulation of plant cell death. Plant Physiol 139: 1914-1926.

Klopffleisch K, Phan N, Augustin K, et al. (2011) Arabidopsis G-protein interactome reveals connections to cell wall carbohydrates and morphogenesis. Mol System Biol 7: 532.

Konishi M, Sugiyama M (2006) A novel plant-specific family gene, *ROOT PRIMORDIUM DEFECTIVE 1*, is required for the maintenance of active cell proliferation. Plant Physiol 140: 591-602.

Lackman P, González-Guzmán M, Tilleman S, et al. (2011) Jasmonate signaling involves the abscisic acid receptor PYL4 to regulate metabolic reprogramming in Arabidopsis and tobacco. Proc Natl Acad Sci U S A 108: 5891-5896.

Lee KW, Cha JY, Kim KH, Kim YG, Lee BH, Lee SH (2012) Overexpression of alfalfa mitochondrial HSP23 in prokaryotic and eukaryotic model systems confers enhanced tolerance to salinity and arsenic stress. Biotechnol Lett 34: 167-174.

Loyola J, Verdugo I, González E, Casaretto JA, Ruiz-Lara S (2012) Plastidic isoprenoid biosynthesis in tomato: physiological and molecular analysis in genotypes resistant and sensitive to drought stress. Plant Biol (Stutt) 14: 149-156.

Muñiz García MN, Giammaria V, Grandellis C, Téllez-Iñón MT, Ulloa RM, Capiati DA (2012) Characterization of StABF1, a stress-responsive bZIP transcription factor from *Solanum tuberosum* L. that is phosphorylated by StCDPK2 *in vitro*. Planta 235: 761-778.

Ouyang B, Yang T, Li H, Zhang L, Zhang Y, Zhang J, Fei Z, Ye Z (2007) Identification of early salt stress response genes in tomato root by suppression subtractive hybridization and microarray analysis. J Exp Bot 58: 507-520.

Pajerowska KM, Parker JE, Gebhardt C (2005) Potato homologs of *Arabidopsis thaliana* genes functional in defense signaling--identification, genetic mapping, and molecular cloning. Mol Plant-Microb Interact 18: 1107-1119.

Peterson FC, Burgie ES, Park SY, Jensen DR, Weiner JJ, Bingman CA, Chang CE, Cutler SR, Phillips GN Jr, Volkman BF (2010) Structural basis for selective activation of ABA receptors. Nature Struct Mol Biol 17: 1109-1113.

Ravet K, Touraine B, Boucherez J, Briat JF, Gaymard F, Cellier F (2009) Ferritins control interaction between iron homeostasis and oxidative stress in Arabidopsis. Plant J 57: 400-412.

Reymond P, Kunz B, Paul-Pletzer K, Grimm R, Eckerskorn C, Farmer EE (1996) Cloning of a cDNA encoding a plasma membrane-associated, uronide binding phosphoprotein with physical properties similar to viral movement proteins. Plant Cell 8: 2265-2276.

Santisree P, Nongmaithem S, Vasuki H, Sreelakshmi Y, Ivanchenko MG, Sharma R (2011) Tomato root penetration in soil requires a coaction between ethylene and auxin signaling. Plant Physiol 156: 1424-1438.

Schneider A, Salamini F, Gebhardt C (1997) Expression patterns and promoter activity of the cold-regulated gene *ci21A* of potato. Plant Physiol 113: 335-345.

Strassner J, Fürholz A, Macheroux P, Amrhein N, Schaller A (1999) A homolog of old yellow enzyme in tomato. Spectral properties and substrate specificity of the recombinant protein*.* J Biol Chem 274: 35067-35073.

Stulemeijer IJ, Stratmann JW, Joosten MH (2007) Tomato mitogen-activated protein kinases LeMPK1, LeMPK2, and LeMPK3 are activated during the Cf-4/Avr4-induced hypersensitive response and have distinct phosphorylation specificities. Plant Physiol 144: 1481-1494.

Taji T, Seki M, Yamaguchi-Shinozaki K, Kamada H, Giraudat J, Shinozaki K (1999) Mapping of 25 drought-inducible genes, *RD* and *ERD*, in *Arabidopsis thaliana*. Plant Cell Physiol 4:, 119-123.

Tian Z-D, Zhang Y, Liu J, Xie C-H (2010) Novel potato C2H2-type zinc finger protein gene, *StZFP1*, which responds to biotic and abiotic stress, plays a role in salt tolerance. Plant Biol (Stutt) 12: 689–697.

Yoshida T, Fujita Y, Sayama H, Kidokoro S, Maruyama K, Mizoi J, Shinozaki K, Yamaguchi-Shinozaki K (2010) AREB1, AREB2, and ABF3 are master transcription factors that cooperatively regulate ABRE-dependent ABA signaling involved in drought stress tolerance and require ABA for full activation. Plant J 61: 672-685.

van't Klooster JW, Kamoun S, Vleeshouwers VGAA, Govers F (1999) The electronic plant gene register. Plant Physiol 121: 1383-1385.

Vasquez-Robinet C, Mane SP, Ulanov AV, et al. (2008) Physiological and molecular adaptations to drought in Andean potato genotypes. J Exp Bot 59: 2109-2123.

Vieten A, Vanneste S, Wisniewska J, Benková E, Benjamins R, Beeckman T, Luschnig C, Friml J (2005) Functional redundancy of PIN proteins is accompanied by auxin-dependent cross-regulation of *PIN* expression. Development 132: 4521-431.

Wang J, Andersson-Gunnerås S, Gaboreanu I, et al. (2011) Reduced expression of the *SHORT-ROOT* gene increases the rates of growth and development in hybrid poplar and Arabidopsis. PLoS ONE 6: e28878.

Wolters AM, Uitdewilligen JG, Kloosterman BA, Hutten RC, Visser RG, van Eck HJ (2010) Identification of alleles of carotenoid pathway genes important for zeaxanthin accumulation in potato tubers. Plant Mol Biol 73: 659-671.

Wu G, Shah DM (1995) Isolation and characterization of a potato catalase cDNA (GenBank U27082) (PGR95-037). Plant Physiol 108: 1748.
